# External Use of Chloral Hydrate

**Published:** 1885-09

**Authors:** 


### External Use of Chloral Hydrate.

Nicolăi has obtained excellent results in the treatment of night sweats, in phthisis and other diseases, by the use of chloral hydrate externally, as follows:

| | | |
|---|---|---|
| ℞ | Chloral Hydrate, - - | dr. 2. |
| | Brandy, - - - - | |
| | Water, - - - | aa oz. 4. |

M. Sig. Lotion.

The patient is sponged off with this lotion every night, and if this does not effect a cure, a shirt should be soaked in the solution and dried, which the patient is to wear at night. This remedy is especially valuable in treating the night sweats of children. Sometimes, three or four applications of this lotion will entirely relieve cases that have been troubled with night sweats for several weeks.—*Paris Medical.*

